# Slicing Through the Options: A Systematic Review of Esophageal Leiomyoma Management

**DOI:** 10.7759/cureus.81614

**Published:** 2025-04-02

**Authors:** Shelleen Gowrie, Anniesha Noel, Candace Wooten, Jennifer Powel, Jerzy Gielecki, Anna Zurada, Michael Montalbano, Marios Loukas

**Affiliations:** 1 Anatomical Sciences, St. George’s University, School of Medicine, St. George's, GRD; 2 Pediatric Medicine, AdventHealth for Children, Orlando, USA; 3 Family Medicine, Optum, New Albany, USA; 4 Obstetrics and Gynecology, Hackensack Meridian Medical Group, Neptune, USA; 5 Anatomy, School of Medicine, University of Warmia and Mazury, Olsztyn, POL; 6 Anatomy/Radiology/Medicine, University of Warmia and Mazury, Olsztyn, POL; 7 Anatomical Sciences, St. George's University, School of Medicine, St. George's, GRD; 8 Anatomy, Nicolaus Copernicus Superior School, College of Medical Sciences, Olsztyn, POL; 9 Clinical Anatomy, Mayo Clinic, Rochester, USA; 10 Pathology, St. George's University, School of Medicine, St. George's, GRD

**Keywords:** esophageal neoplasms, minimally invasive surgical procedures, robot-assisted thoracoscopic surgery, submucosal tunneling endoscopic resection, treatment outcomes, video-assisted thoracoscopic surgery (vats)

## Abstract

Esophageal leiomyomas are rare, benign tumors that can remain asymptomatic or cause dysphagia and chest discomfort when they grow large. Despite advancements in diagnostic and therapeutic strategies, optimal management remains debated. This systematic review evaluates current diagnostic modalities and treatment approaches, synthesizing findings from a comprehensive PubMed search following the Preferred Reporting Items for Systematic Reviews and Meta-Analyses (PRISMA) guidelines. A total of 51 studies were included, comprising six original studies, 26 case reports, nine retrospective cohort studies, nine case series, and two cross-sectional studies.

Findings indicate that endoscopic ultrasonography (EUS) is the most accurate diagnostic tool (89% accuracy), while computed tomography (CT) and barium swallow studies provide complementary structural assessments. Immunohistochemical staining differentiates leiomyomas from gastrointestinal stromal tumors (GISTs), with leiomyomas expressing desmin and smooth muscle actin (SMA) but lacking CD34 and KIT.

Surgical intervention is recommended for symptomatic tumors or those exceeding 5 cm. Minimally invasive techniques, including robotic-assisted thoracoscopic surgery (RATS) and submucosal tunneling endoscopic resection (STER), offer superior outcomes compared to traditional open surgery. RATS demonstrates a negligible mucosal injury rate versus 1-15% for other approaches, while STER minimizes blood loss and accelerates recovery. Postoperative outcomes are generally favorable, though transient gastroesophageal reflux disease (GERD) is the most common complication.

While STER and RATS present effective alternatives with reduced morbidity, this review highlights limitations, including variability in study designs, small sample sizes, and a lack of long-term follow-up data. Further prospective studies are needed to optimize patient selection and establish long-term efficacy. This review provides insights to inform clinical practice and guide future research in the management of esophageal leiomyomas.

## Introduction and background

Esophageal leiomyomas are rare, benign, and slow-growing mesenchymal tumors originating from the muscularis propria layer and, less frequently, from the muscularis mucosa [1-3]. First identified by Sussius in 1559, esophageal leiomyomas were later recognized as a distinct gastrointestinal neoplasm by Giovanni Morgagni in 1761. Over time, key contributions from surgeons and pathologists such as Alexander Munro, Virchow, Sauerbruch, Ohsawa, and Churchill have led to our current understanding of the pathology, classification, and treatment of esophageal leiomyoma [4,5].

**Figure 1:**
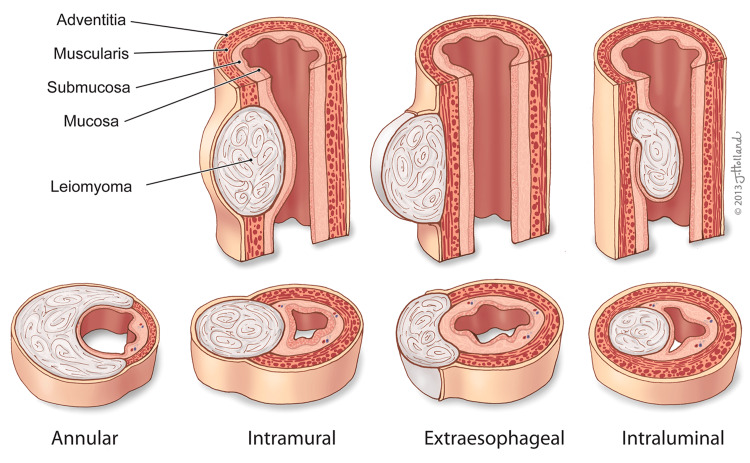
Classification of esophageal leiomyomas based on growth patterns. A diagram illustrating the classification of esophageal leiomyomas based on their growth patterns (intraluminal, extraluminal, and mixed). Figure Credit: This original illustration was created by Jessica Holland, MSMI, CMI, for the authors. This image was created using traditional drawing tools and then later rendered in Adobe Photoshop and labeled in Adobe Illustrator (© CC-BY-ND 2025 Jessica Holland, MSMI, CMI, St. George's University).

As the most common benign esophageal neoplasm, esophageal leiomyomas are often classified based on histology or location [1]. Histologically, esophageal leiomyomas are composed of spindle-shaped smooth muscle cells without atypia or necrosis. Based on location and growth pattern, esophageal leiomyomas fall into four primary categories: intramural, intraluminal, extraesophageal, and annular (Figure 1) [1-5].

A total of 62.9% of esophageal leiomyomas are intramural, originating from the muscularis mucosa, while the remaining cases arise from the muscularis propria [6]. The rarest subtype is the annular or horseshoe-shaped leiomyoma, which accounts for only 2% of cases [7]. The increased use of EUS has improved the detection and classification of these tumors, providing greater insight into their growth patterns and anatomical distribution within the esophagus.

**Figure 2:**
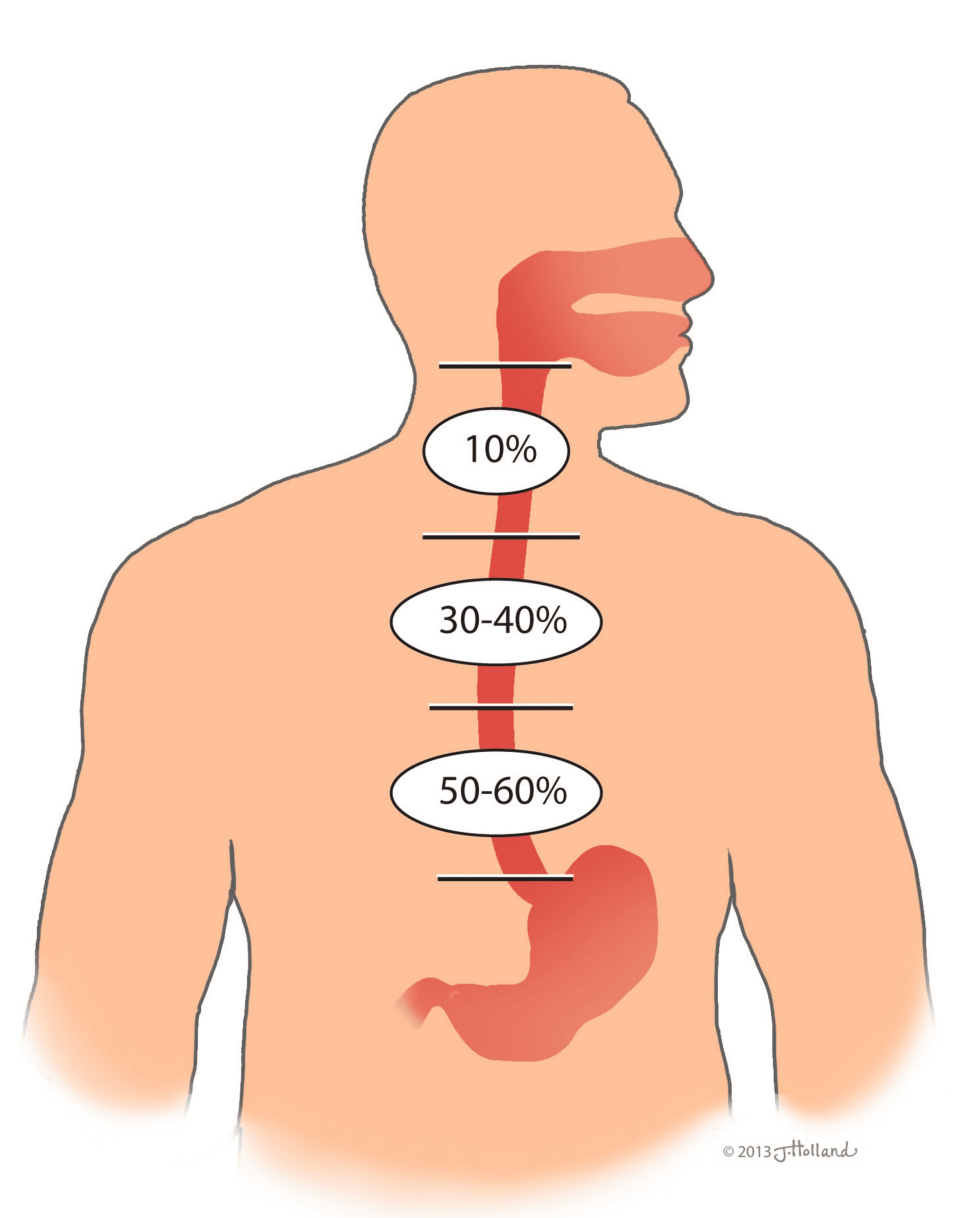
Distribution of esophageal leiomyomas based on anatomical location. A diagram illustrating the distribution of esophageal leiomyomas based on their anatomical location (upper, middle, and lower third of the esophagus). Figure Credit: This original illustration was created by Jessica Holland, MSMI, CMI, for the authors. This image was created using traditional drawing tools and then later rendered in Adobe Photoshop and labeled in Adobe Illustrator (© CC-BY-ND 2025 Jessica Holland, MSMI, CMI, St. George's University).

Most often, esophageal leiomyomas are classified by their location within the esophagus, with the majority (50-60%) found in the lower third, followed by 30-40% in the middle third and 10% in the upper third (Figure 2) [1-3]. This distribution corresponds to the increasing presence of smooth muscle cells in the distal esophagus. While traditional imaging techniques such as X-ray, CT, and MRI can identify these tumors, endoscopic ultrasound (EUS) has significantly enhanced their characterization, particularly in identifying their layer of origin within the esophageal wall.

Most esophageal leiomyomas are asymptomatic and incidentally discovered; however, larger tumors may cause dysphagia, chest pain, retrosternal discomfort, and, in rare cases, tracheal compression leading to respiratory distress [8-10]. Their true incidence remains difficult to establish, as smaller, asymptomatic lesions often go undetected. Reported incidences vary widely, ranging from 0.005% to 5.1%, depending on the population studied [11-13]. While usually solitary, multiple leiomyomas have been reported in 3-4% of patients [14]. Although esophageal leiomyomas may develop at any age, 90% occur between the ages of 20 and 69 years, with a peak incidence between the ages of 20 and 50 years [1,2,5]. In adults, men are more commonly affected than women, with a ratio of approximately 2:1 [2,4,15].

Advances in imaging and minimally invasive surgery have significantly transformed the management of esophageal leiomyomas. Traditional imaging techniques such as X-ray, CT, and MRI have been valuable for identifying these tumors, but EUS has emerged as the gold standard for precise characterization of their layer of origin [16]. While thoracotomy with enucleation has long been the standard surgical approach for symptomatic cases, newer techniques such as video-assisted thoracoscopic surgery (VATS), submucosal tunneling endoscopic resection (STER), and robotic-assisted thoracoscopic surgery (RATS) offer less invasive alternatives with improved patient outcomes.

Despite these advancements, the optimal management strategy for esophageal leiomyomas remains debated, particularly regarding when to intervene surgically versus monitoring asymptomatic cases. While several case reports and retrospective studies have described individual experiences with esophageal leiomyoma management, there is no recent systematic review synthesizing the existing literature on diagnosis and treatment outcomes. This study aims to bridge that gap by providing a comprehensive review of current treatment options, their outcomes, and evolving trends in esophageal leiomyoma management. By synthesizing findings from multiple studies, this review seeks to inform medical understanding and highlight areas for future research.

## Review

Materials and methods

Search Strategy

A systematic literature search was conducted by two independent researchers in the electronic database PubMed for studies published up to June 27th, 2024, strictly adhering to the Preferred Reporting Items for Systematic Reviews and Meta-Analyses (PRISMA) guidelines. The keywords used to identify relevant studies were as follows: “esophageal leiomyoma” and “leiomyoma of the esophagus”. To ensure data integrity, duplicates were removed by comparing the lists obtained from PubMed using Excel (Microsoft Corporation, Redmond, WA).

Selection Criteria

The inclusion criteria were as follows: (1) clinical studies discussing esophageal leiomyoma; and (2) full-text articles that reported necessary data for statistical analysis. The exclusion criteria were as follows: (1) studies that were letters to the editor; (2) involved non-human subjects; (3) were not written in English; or (4) involved esophageal leiomyoma with overlying carcinoma.

The search was conducted without temporal restrictions, including studies published from 1946 to 2024, as available on the PubMed database.

Two reviewers, SG and AN, independently screened titles and abstracts and assessed full-text articles for eligibility to minimize bias and ensure a comprehensive review. In cases of disagreement, consensus was reached through discussion between the two reviewers with additional reviewers, MM and ML, as appropriate. Data were extracted from outcomes to provide a comprehensive overview of the diagnosis and treatment of esophageal leiomyoma.

Supplementing our electronic database search, we also conducted a thorough search of authorial collections to identify additional relevant studies. This approach yielded 52 articles through back citation that were not captured in the initial electronic search. These articles were included in the review if, upon retrieval of the full-text article, they met the eligibility criteria and were applicable to the study’s content. All studies were evaluated based on the same inclusion and exclusion criteria applied to the initially identified studies.

Results

Study Identification

**Figure 3:**
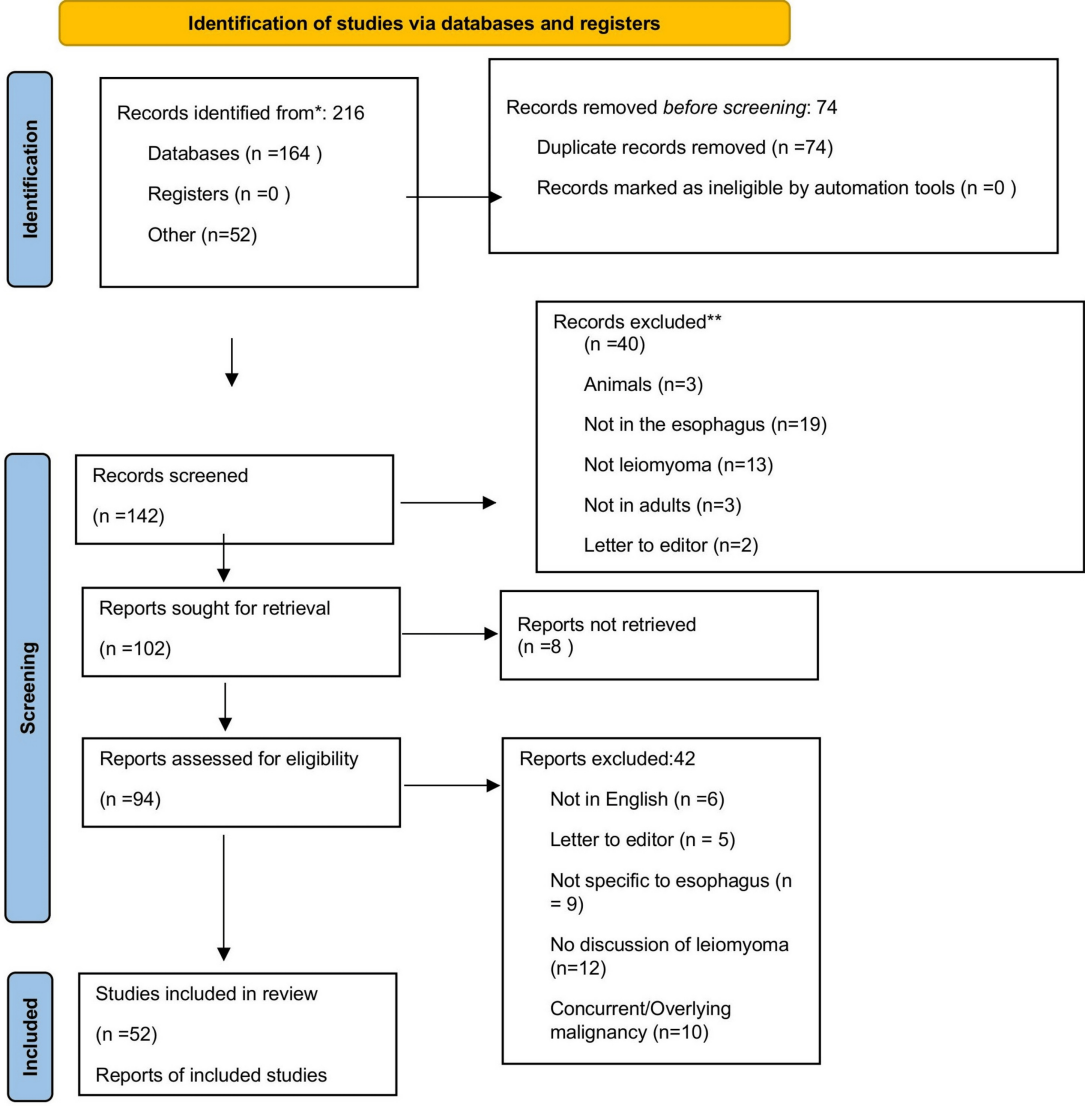
PRISMA diagram of the study selection process. Diagrammatic representation of the study selection process for the systematic review, following the Preferred Reporting Items for Systematic Reviews and Meta-Analyses (PRISMA) guidelines. The flowchart highlights the number of studies identified, screened, assessed for eligibility, and ultimately included in the review.

Thorough database searches and reference reviews identified 216 studies published between 1947 and 2024. Of these, 52 articles were identified through review articles. To ensure data integrity, 74 articles were removed following deduplication. Additionally, 40 articles were removed from the screening based on irrelevant titles or abstracts to exclude three animal studies, 19 non-esophageal localized studies, 13 studies describing other esophageal pathologies, three non-adult studies, and two letters to the editor. Screening and eligibility were performed according to the stated inclusion and exclusion criteria. Screening for full-text retrieval in English led to the exclusion of an additional eight articles. Following the screening of the remaining 92 articles for eligibility at the final screening stage, a total of 42 articles were excluded because they were not in English, were letters to the editor, not specific to the esophagus, or did not discuss leiomyomas or discussed leiomyoma in the context of concurrent or overlying malignancies or pathologies. Ultimately, a total of 52 studies met the eligibility criteria for inclusion. Figure 3 illustrates a flow diagram showing the inclusion process in accordance with the PRISMA guidelines.

Characteristics of Included Studies

A total of 52 studies were included in the review, categorized as follows: six original studies, 26 case reports, nine retrospective cohort studies, nine case series, and two cross-sectional studies (Figure 3).

The inclusion of a broad range of study designs allowed for a comprehensive review of the literature on esophageal leiomyomas. While case reports, comprising the largest portion (n = 26), provide valuable insights into rare and unique presentations, their limitations in terms of generalizability must be acknowledged. Case reports typically involve small sample sizes and lack comparison groups, which restricts the ability to make broad inferences about treatment effectiveness or outcomes in larger populations.

On the other hand, retrospective cohort studies (n = 9), though subject to inherent biases such as recall bias and incomplete data, contribute valuable statistical insights and help establish patterns and correlations within larger cohorts. These studies provide a stronger foundation for evaluating treatment modalities and patient outcomes, though their observational nature means they cannot establish causality.

Thus, while case reports are invaluable for illustrating individual cases, the retrospective cohort strengthens the review by providing statistical context and trend analysis, helping to balance the limitations of case-based evidence.

Esophageal Leiomyoma Characteristics and Epidemiology

Esophageal leiomyomas are the most common benign esophageal neoplasms, predominantly found in the middle and lower thirds of the esophagus, where smooth muscle is most abundant. The tumors are more common in men, with a male-to-female ratio of approximately 2:1, and are most frequently diagnosed in individuals aged 20-50 years. Most leiomyomas are small, slow-growing, and asymptomatic, though larger tumors (>5 cm) can cause symptoms such as dysphagia and chest pain. Studies have shown that leiomyomas account for 70-80% of esophageal subepithelial tumors (SETs) but remain rare overall, with an estimated incidence ranging from 0.002% to 5.9% in autopsy studies. The majority originate from the muscularis mucosa or muscularis propria layers, with solitary lesions being most common (Table 1).

**Table 1:** Esophageal leiomyoma characteristics, classification, and epidemiology. SETs: subepithelial tumors; GISTs: gastrointestinal stromal tumors; EUS: endoscopic ultrasound; STS: Society of Thoracic Surgeons; ESGE: European Society of Gastrointestinal Endoscopy; VATS: video-assisted thoracoscopic surgery.

Study	Key findings
Choong & Meyers (2003) [1]	Esophageal leiomyomas are classified based on histologic cell types and their relationship to the esophageal wall. Most occur in the middle and lower thirds of the esophagus. Five main clinical presentations: asymptomatic cases, intraluminal obstruction, extramural compression, regurgitation of pedunculated tumors, and ulceration/bleeding. USA (Missouri) autopsy prevalence of esophageal leiomyoma ranging from 0.17% to 0.59%. An estimated male-to-female ratio of 2:1 with most cases being diagnosed in the 20-59 years age group.
Mathew et al. (2024) [3]	Slow-growing tumors with low malignant potential are often detected incidentally when less than 5 mm in size. In the USA (New York), it is most common in men (2:1 ratio) and typically diagnosed between the ages of 20 and 50. It is mostly located in the lower two-thirds of the esophagus.
Bogedain et al. (1963) [4]	The first recorded case of esophageal leiomyoma was in 1559 with a description by Sussius. In the USA (Ohio), prevalence is approximately 0.002%, with an estimated male-to-female ratio of 2:1 for distribution, with most cases being described in adults ranging from 20 to 80 years old (peak incidence in the 40s). Interestingly, most post-mortem cases were found in adults in the 7th decade of life, while most operative cases were described in adults in their 40s, with 33% and 45.2% in the middle and lower thirds of the esophagus, respectively.
Lee et al. (2004) [5]	Leiomyomas were first described as distinct GI neoplasms by Morgagni in 1761. In 1797, Munro was the first to identify a localized, intramural leiomyoma of the esophagus. Incidence of leiomyoma might be underestimated due to it possibly being missed on autopsy, but it ranges from 0.005 to 5.1%. A total of 90% of cases have been reported in adults ranging from 20 to 69 years old, with a male-to-female ratio of 2:1 in the USA (Pennsylvania). Approximately 56% and 33% of leiomyomas have been found in the lower and middle 2/3 of the esophagus, respectively.
Lewis et al. (2013) [15]	Leiomyomas are the most common benign esophageal neoplasms. Esophageal leiomyomas typically appear as smoothly marginated intramural lesions that are approximately twice as common in men as in women. They have been found in patients aged 4-81 years old, but rarely occur in the pediatric population in the USA (Chicago). Small, slow-growing tumors are usually less than 30 mm and often found in the lower 2/3 of the esophagus, where smooth muscle in the esophagus is greater.
Postlethwait and Musser (1974) [13]	An autopsy study highlighting incidental findings of esophageal leiomyomas and other structural changes. Of autopsy specimens, 5.1% contained esophageal leiomyomas found post-mortem in the USA (North Carolina). Most of these were solitary lesions, but 9.8% of specimens had multiple lesions, which is a rarer phenomenon. Most esophageal leiomyomas are found in the lower 1/3 of the esophagus (n = 21), followed by the middle third (n = 9).
Xu et al. (2012) [17]	Esophageal leiomyomas account for approximately 1.2% of esophageal tumors in China (Hangzhou), with an age range of 25-80 years and peak incidence between 40 and 60 years. A cohort of 229 cases of esophageal leiomyoma diagnosed using EUS showed solitary lesions in 94.8% of the population, with a predominance in the lower 2/3 of the esophagus, with the majority originating from the muscularis mucosa (78.6%), followed by the muscularis propria (14.4%) in this cohort.
Kang et al. (2022) [18]	Leiomyomas are the most common esophageal subepithelial tumors (SETs) in Korea (Seoul). EUS is the most valuable diagnostic tool for SETs, helping assess size, margins, layer of origin, and echogenicity. However, differentiating benign and malignant SETs, especially distinguishing leiomyomas from GISTs, is challenging, often requiring pathological confirmation.
Rao et al. (2020) [19]	Benign esophageal tumors account for <1% of all esophageal tumors, with leiomyomas making up two-thirds. In China (Jilin Province), esophageal leiomyomas less than 50 mm were incidentally found and were more common in middle-aged males (2:1 male to female ratio). Leiomyomas <50 mm are typically asymptomatic, while larger tumors can cause dysphagia and chest pain.
Deprez et al. (2022) [20]	ESGE recommends against the surveillance of asymptomatic esophageal leiomyomas. Leiomyomas are most commonly found in the lower 2/3 of the esophagus, often originating from the muscularis propria layer.
Little et al. (2014) [21]	Esophageal leiomyomas are rare, benign, slow-growing tumors. These tumors are small, typically less than 50 mm, and have a very low potential for malignant transformation. The Society of Thoracic Surgeons (STS) suggests a conservative approach for asymptomatic leiomyomas and favors minimally invasive techniques like VATS when surgery is necessary.
Punpale et al. (2007) [22]	Esophageal leiomyomas are rare, benign, slow-growing tumors that account for less than 1% of esophageal neoplasms in India (Mumbai). They are the most common benign mesenchymal tumors of the esophagus, accounting for 2/3 of all benign lesions in the esophagus. Typically detected as intramural growths less than 50 mm in the lower two-thirds of the esophagus.
Kernstine et al. (2009) [23]	For tumors located in the upper two-thirds of the esophagus and more than 40 to 50 mm above the gastroesophageal junction, open enucleation via right thoracotomy has long been the standard procedure.
Zwischenberger et al. (2001) [24]	Tumors in the lower third of the esophagus may also require an anti-reflux procedure with enucleation or resection.
Standards of Practice Committee (2017) [25]	Esophageal leiomyomas are benign tumors of smooth muscle that commonly originate from the muscularis mucosa or the muscularis propria. A total of 90% of esophageal leiomyomas are found in the lower 2/3 of the esophagus. Asymptomatic patients do not require any intervention, including endoscopic surveillance or follow-up, unless symptomatic.
Conca et al. (2023) [26]	Most common benign intramural tumor of the esophagus, though still rare. Usually solitary, with multiple lesions being very rare. More common in men, with an average age of 44 years in Argentina. Primarily located in the middle and distal thirds, where smooth muscle is most abundant. A total of 4.5-23% of cases are associated with hiatal hernia.
Asaf et al. (2022) [27]	A retrospective cohort of 11 cases of esophageal leiomyoma in India (New Delhi). It is the most common benign esophageal tumor, accounting for 70% of all benign esophageal tumors. Characterized as benign, slow-growing, smooth muscle tumors are often found in male adults ranging from 20 to 69 years old, with the peak incidence in the 50s. Most are located in the middle and lower third of the esophagus, 30% and 60%, respectively.
A-Lai et al. (2022) [28]	Esophageal leiomyomas account for 70-80% of submucosal tumors in the esophagus and less than 1% of all neoplasms in the esophagus in China (Chengdu). Usually found incidentally in the lower two-thirds of the esophagus in patients ranging from 20 to 50 years old, with a male-to-female ratio of 2:1.
Karagülle et al. (2008) [29]	Leiomyomas are slow-growing, oval or spherical masses that occur in all parts of the esophagus with the following distribution: 60% in the distal third, 30% in the middle, and 10% in the proximal esophagus. They are often intramural, with approximately half of the patients being asymptomatic (49% reported to be less than 50 mm). Autopsy incidence in Turkey (Konya) is estimated to be between 0.005% and 5.1%, with peak incidence reported in middle-aged males.

Clinical Presentation and Symptoms

Among the included studies, leiomyomas were often found incidentally during imaging or autopsy. Tumors smaller than 50 mm were frequently asymptomatic, while larger tumors presented with dysphagia, retrosternal discomfort, heartburn, and regurgitation. Less commonly, patients experienced respiratory symptoms, such as dyspnea and cough, due to extrinsic airway compression. In rare cases, giant leiomyomas have led to severe complications, including sudden asphyxial death. Case reports highlighted a spectrum of clinical presentations, from incidental findings to life-threatening airway obstruction (Table 2).

**Table 2:** Esophageal leiomyoma clinical presentation and symptoms. GERD: gastroesophageal reflux disease.

Study	Study design	Key findings
Choong & Meyers (2003) [1]	Literature review	Symptoms are categorized into obstruction, extramural compression, regurgitation, ulceration, or asymptomatic cases.
Mathew et al. (2024) [3]	Literature review	Tumors <50 mm are usually asymptomatic. Larger tumors may cause dysphagia, chest pain, retrosternal discomfort, heartburn, and regurgitation.
Jones & Sweet (1947) [8]	Case report	Post-mortem diagnosis of esophageal leiomyoma in a patient with dysphagia.
Peacock et al. (1985) [10]	Case report	Rare case of sudden asphyxia due to an esophageal leiomyoma, highlighting airway compression as a severe but uncommon presentation.
Punpale et al. (2007) [22]	Retrospective cohort	The most common symptom was dysphagia, followed by non-ulcerative dyspepsia/GERD, retrosternal burning, and epigastric abdominal pain. Rare symptoms include dyspnea, cough, and hoarseness secondary to mass effect due to obstruction by a large esophageal leiomyoma mass.
Conca et al. (2023) [26]	Case series	The most common symptom is dysphagia, followed by retrosternal pain, pyrosis, and discomfort. Other symptoms include nausea, vomiting, reflux, breathing problems, and bleeding. Some cases are asymptomatic.
Asaf et al. (2022) [27]	Retrospective cohort	Mostly asymptomatic, with symptomatic cases presenting with varying severity of dysphagia, retrosternal pain, epigastric pain, cough, and dyspnea.
A-Lai et al. (2022) [28]	Retrospective cohort	Patients are mostly asymptomatic. However, symptomatic cases present with varying severity of dysphagia, retrosternal pain, and epigastric discomfort. Presentation of a single esophageal leiomyoma in the lower two-thirds of the esophagus is more common than multiple leiomyomas.
Karagülle et al. (2008) [29]	Case report	The most common symptom is typically dysphasia, followed by retrosternal pain. Bleeding and weight loss are rare symptoms.
Sun et al. (2017) [6]	Cross-sectional	Among 167 confirmed cases, 48 (28.7%) were diagnosed unexpectedly during medical checkups. Among 119 symptomatic patients, the most common symptom was upper abdominal discomfort (40.1%). Acid reflux and heartburn occurred in 9.0% (15/119), and dysphagia was also reported in 9.0% (15/119). Other symptoms included thoracalgia (6.6%), dyspepsia (4.2%), paresthesia of the pharynges (1.8%), and nausea (0.6%).
Yalçınkaya et al. (2020) [30]	Retrospective cohort	The most common symptom was dysphagia (46.2%), followed by chest pain. Of the cohort, 38.5% were asymptomatic and diagnosed incidentally.
Rege et al. (2023) [31]	Case series	Retrosternal burning pain was the most common symptom in lower esophageal tumors, while middle-third tumors were often asymptomatic and detected incidentally. Other symptoms reported among the 19 cases in a retrospective cohort included dysphagia (n = 5), melena (n = 1), and weight loss (>5% body weight in three months, n = 4). Symptom duration ranged from one to 24 months.
Kemuriyama et al. (2021) [32]	Case report	Patients with esophageal leiomyoma are usually asymptomatic. However, symptomatic patients can present with chest pain, dyspnea, and/or upper abdominal pain.
Elliott et al. (2021) [33]	Case report	Patients are often asymptomatic. Can present with vague epigastric discomfort, dysphagia, and/or odynophagia.

Diagnosis and Imaging

Multiple diagnostic modalities have been used for identifying esophageal leiomyomas. EUS was found to be the most effective tool, offering an 89% diagnostic accuracy by visualizing tumor size, margins, layer of origin, and echogenicity. EUS-guided fine-needle aspiration (FNA) was useful for histological confirmation but had limitations in cases where deep tissue sampling was required. Computed tomography (CT) and barium swallow studies provided additional diagnostic insights, particularly for larger tumors. CT typically revealed homogeneously attenuated masses with occasional calcifications, while barium swallow studies showed smooth, crescent-shaped filling defects. Immunohistochemical (IHC) staining was critical for differentiation from malignant gastrointestinal stromal tumors (GISTs), as leiomyomas were consistently positive for desmin and smooth muscle actin (SMA) but negative for CD34 and KIT (Table 3).

**Table 3:** Diagnosis and imaging in esophageal leiomyoma. EUS: endoscopic ultrasonography; SETs: subepithelial tumors; CT: computed tomography; EUS-FNAB: endoscopic ultrasound-guided fine needle aspiration and biopsy; EUS-FNA: endoscopic ultrasound-guided fine needle aspiration; UGI endoscopy: upper gastrointestinal endoscopy; EGD: esophagogastroduodenoscopy; MRI: magnetic resonance imaging; SMA: smooth muscle actin; GIST: gastrointestinal stromal tumor; GCT: granular cell tumor; IHC: immunohistochemistry.

Study	Study design	Sample size	Main findings
Mathew et al. (2024) [3]	Literature review	Not applicable	Often diagnosed incidentally. Upper GI barium contrast study is a simple and widely available diagnostic tool.
Lee et al. (2004) [5]	Literature review	Not applicable	Chest X-ray: rounded, lobulated, homogenous mass with possible calcifications demonstrating lateral growth in the mediastinum. Barium swallow: smooth, spherical, or lobulated filling defect in the esophagus with sharp demarcation between the tumor edge and the unaffected esophagus. EUS: homogenously hypoechoic, well-circumscribed mass with a smooth outer border originating from one of the five visualized layers comprising the esophageal wall. CT: smooth, marginated, homogenous, low or iso-attenuated mass. A definitive diagnosis can only be made by histologic examination; however, intraoperative biopsy is contraindicated.
Lewis et al. (2013) [15]	Case series	23	Radiological and pathological correlation of esophageal neoplasms, detailing the imaging features of leiomyomas. Chest X-ray: mediastinal masses with abnormal contour of the azygoesophageal line and coarse calcifications occasionally. Barium swallow: smooth-surfaced, crescent-shaped filling defects that form right angles or slightly obtuse angles with the adjacent esophageal wall or short strictures if the leiomyoma encircles the esophagus. CT/MRI: smoothly marginated, homogenous mass with occasional calcifications that are hypo/isoattenuating on CT or slightly hyperintense on MRI. EUS: 89% diagnostic accuracy. Homogeneously, hypoechoic mass arising from one of the five visualized layers of the GI wall. Most commonly, the muscularis mucosa or propria layers.
Xu et al. (2012) [17]	Case series	229	Endoscopic ultrasonography (EUS) plays a crucial role in diagnosis and treatment selection, being able to visualize the five layers of the gastrointestinal wall and provide information regarding the nature, size, number, and originating layers of lesions. A total of 229 patients were diagnosed with EUS; postoperative findings showed a diagnostic accuracy of 88.6%, with 110 patients' postoperative histology being consistent with preoperative EUS diagnosis.
Kang et al. (2022) [18]	Case series	262	EUS is valuable for assessing SETs but has a lower diagnostic accuracy for lesions <30 mm. EUS-FNAB is the most commonly used tissue sampling method, with an 80% diagnostic yield (20/25 cases), though some samples remain non-diagnostic. Immunohistochemistry staining for leiomyomas showed positivity for smooth muscle actin (SMA) and desmin, while GISTs were CD34 and KIT (c-KIT) positive, aiding in differentiation. Pathological confirmation is often necessary for a definitive diagnosis.
Rao et al. (2020) [19]	Case report	1	CT and EUS can diagnose most upper GI leiomyomas, but atypical lesions may mimic stromal tumors. EUS-FNA can help differentiate leiomyomas from malignant gastrointestinal stromal tumors (GISTs). Large symptomatic leiomyomas require resection, but smaller lesions are managed with regular follow-up. Histopathology: leiomyomas consist of intersecting spindle cells with rich cytoplasm and low malignant potential. IHC markers: desmin and SMA positive; CD34 and CD117 negative, which helps differentiate leiomyomas from GISTs. EUS-FNA is crucial for confirming diagnosis, especially when lesions resemble stromal tumors on CT.
Punpale et al. (2007) [22]	Retrospective cohort	6	Barium swallow: smooth, concave mass with intact underlying mucosa with distinct, sharp angles at the junctions of the tumor and normal surrounding tissue. EUS: homogenous, hypoechoic mass.
Kernstine et al. (2009) [23]	Case report	1	Endoscopy: mobile, smooth, round, raised submucosal mass with intact overlying intraluminal mucosa.
Standards of Practice Committee(2017) [25]	Guideline	Not applicable	EUS: well-circumscribed, hypoechoic mass typically found in the 2nd, 3rd, or 4th layers of the esophageal wall.
Conca et al. (2023) [26]	Case series	9	Pneumo-CT shows leiomyomas as smooth or slightly lobulated masses, occasionally with calcifications. Rarely, they may present with cystic degeneration, necrosis, or ulceration. They have homogeneous attenuation with a scant-to-moderate enhancement pattern on contrast imaging. Histologically, leiomyomas consist of spindle-shaped cells with elongated nuclei and eosinophilic cytoplasm, often with calcifications. They are positive for desmin and smooth muscle markers but negative for CD34 and KIT, distinguishing them from GISTs. Immunohistochemistry is crucial for differentiation, as leiomyomas do not metastasize, unlike GISTs. Surgical resection is not necessary unless symptomatic.
Asaf et al. (2022) [27]	Retrospective cohort	12	Diagnosed preoperatively using EUS, CT, and UGI endoscopy. Noted to have a well-defined, smooth, hypoechoic appearance in EUS.
Karagülle et al. (2008) [29]	Case report	1	Chest X-ray: posterior or mediastinal mass. Barium swallow: smooth, crescent-shaped filling defect in the esophageal lumen without the presence of a mucosal abnormality. CT: weekly, homogenous contrast enhancement. MRI: isointense submucosal lesion. EUS: homogenous, hypoechoic mass with clear margins with a hyperechoic border.
Sun et al. (2017) [6]	Cross-sectional	225	Two hundred and twenty-five patients with suspected esophageal leiomyomas on EUS were examined. One hundred and sixty-seven were confirmed by pathology (91.75%). Misdiagnosed cases included mesenchymoma, inflammatory mass, schwannoma, or esophageal carcinoma. Of leiomyomas, 62.9% originated from the muscularis mucosa, and the rest from the muscularis propria. EUS showed 86.8% as homogeneous echo lesions. Morphologies included spherical (79.6%), fusiform (9.6%), polypoidal (0.6%), and irregular (10.2%). Ten patients had exophytic lesions (6.0%). Leiomyomas from the muscularis mucosa were smaller and closer to the incisors than those from the muscularis propria (p < 0.05). Patients preferred endoscopic therapy, which resulted in fewer adverse events compared to surgery (p < 0.05). Superficial leiomyomas had fewer complications and better recovery (p < 0.05). Median hospitalization was 8 ± 6 days, and post-treatment hospitalization was 4 ± 3 days. Compared with CT, EUS was superior for detecting leiomyomas, especially those from the muscularis propria with large diameters or inhomogeneous echoes.
Rege et al. (2023) [31]	Case series	19	CT and EUS were used to diagnose esophageal leiomyoma in all 19 cases. CT scans revealed well-defined, homogeneously attenuated masses arising from the esophagus with rare instances of calcifications and absent lymphadenopathy. EUS demonstrated hypoechoic lesions arising from the muscularis propria.
Elliott et al. (2021) [33]	Case report	1	Appears as a smooth-walled, well-circumscribed mass arising from the fourth layer of the esophagus on EUS.
Schaefer et al. (2022) [34]	Narrative review	Not applicable	EUS alone is not sufficient to definitively diagnose because it is difficult to distinguish from malignant tumors like GIST.
Ryu et al. (2022) [35]	Retrospective cohort	45	Comparison between esophageal leiomyomas and granular cell tumors (GCTs), the two most common subepithelial tumors. In a study of 11 leiomyomas and 38 GCTs, leiomyomas were predominantly located in the upper third of the esophagus (45.5%). EUS revealed that leiomyomas had echogenicity similar to proper muscle and a significantly clearer hyperechoic epithelial lining compared to GCTs (100% vs. 26.7%, p < .001). Endoscopic forceps biopsy was often inadequate for definitive diagnosis due to the subepithelial location, with aggressive tissue sampling posing a risk of hemorrhage. Increasing endoscopic screening in South Korea has led to more incidental detections of asymptomatic esophageal subepithelial tumors.

Treatment Approaches and Surgical Outcomes

The management of esophageal leiomyomas depended on tumor size, location, and symptom severity. Asymptomatic tumors smaller than 5 cm were often managed conservatively with periodic EUS monitoring, as recommended by the European Society of Gastrointestinal Endoscopy (ESGE). However, surgical intervention was indicated for symptomatic patients or tumors exceeding 50 mm.

Surgical approaches included VATS, RATS, STER, and open thoracotomy. VATS and RATS were associated with shorter hospital stays, reduced postoperative pain, and lower complication rates compared to thoracotomy. RATS showed advantages in preventing mucosal injuries, with studies reporting a 0% mucosal injury rate compared to 1-15% for VATS and open surgery.

STER emerged as a minimally invasive option with high success rates for small tumors. Studies demonstrated that STER resulted in shorter operative times, reduced blood loss, and faster postoperative recovery compared to VATS. However, thoracotomy remained necessary for large tumors encircling the esophagus or causing significant extrinsic compression. Segmental esophageal resection was performed in rare cases involving giant leiomyomas with airway obstruction.

Outcomes across the reviewed studies were favorable, with minimal postoperative complications and no tumor recurrence reported at follow-ups ranging from three months to six years. The most common postoperative issues included transient gastroesophageal reflux disease (GERD), which was managed effectively with proton pump inhibitors (PPIs) (Table 4).

**Table 4:** Surgical management and treatment outcomes in esophageal leiomyoma. VATS: video-assisted thoracoscopic surgery; RATS: robotic-assisted thoracoscopic surgery; STER: submucosal tunneling endoscopic resection; MIS: minimally invasive surgery; P-STER: piecemeal submucosal tunneling endoscopic resection; SMA: smooth muscle actin; CXR: chest X-ray; GERD: gastroesophageal reflux disease; PPI: proton pump inhibitors.

Study	Approach	Outcomes
Choi et al. (2011) [2]	Thoracotomy vs. video-assisted thoracoscopic surgery (VATS)	VATS was more frequently used in females, asymptomatic patients, and upper-third lesions. The VATS group had a shorter hospital stay (8.0 vs. 10.7 days, p = 0.006), with no perioperative deaths and minimal complications.
Luh et al. (2012) [11]	Video-assisted thoracoscopic surgery (VATS)	12 patients underwent VATS for esophageal leiomyoma. Eight patients underwent a right-sided VATS approach for tumors located in the upper two-thirds of the esophagus. The other four with esophageal tumors located in the lower third of the esophagus underwent a left-sided VATS approach. Median operative time was 95 minutes (70-230 minutes). No major complications such as death, leaks, mucosal tears, or other complications. No recurrence or complications at follow-ups at 12 and 98 months postoperatively.
Rao et al. (2020) [19]	Laparoscopic local resection	80 × 60 × 35 mm tumor with desmin (+), SMA (+), CD34 (-), CD117 (-), H-caldesmon (+), Ki-67 (+1%), SDHB (+), Dog-1 (-), and S-100 (-) immunohistochemistry indicative of leiomyoma confirmed after resection. The patient recovered well postoperatively with no complications.
Punpale et al. (2007) [22]	Esophageal resection of giant leiomyomas	Segmental esophageal resection is indicated for large leiomyomas, with total esophagectomy being appropriate in cases involving long segments of the esophagus. Three out of six patients required esophageal resection. Of the other three, two underwent local enucleation, and one asymptomatic patient with a small tumor was observed. The patients who underwent esophageal resection had large tumors that extrinsically compressed surrounding structures such as the tracheobronchial tree. All operated patients had uneventful postoperative recovery, apart from the patient who underwent total transthoracic esophagectomy, who had a minor anastomotic leak in the neck, which resolved with conservative management. All patients were asymptomatic and had no recurrence at follow-up.
Asaf et al. (2022) [27]	Robotic-assisted thoracoscopic (RATS) enucleation	11 cases of RATS with no conversions. One case of delayed pericardial tamponade. Overall good postoperative recovery with patients being discharged post-op day one, if no complications. Oral gastrografin swallow the next morning, liquid diet for four days, semi-solid diet after one week. CXR at discharge, six-monthly follow-ups for two years, then annual follow-ups for five years.
A-Lai et al. (2022) [28]	Thoracoscopy vs. thoracotomy for esophageal leiomyoma enucleation	10 patients underwent thoracotomy, while 24 underwent thoracoscopic enucleation. Thoracoscopy resulted in smaller incisions, reduced trauma, shorter hospital stays, and less postoperative pain. However, thoracotomy was required for cases with complete esophageal encirclement by the tumor (p = 0.002) or requiring the left lateral surgical approach (p = 0.001). Tumor size alone was not a risk factor for thoracotomy. No perioperative deaths. Follow-up over 63.5 months showed no recurrence or mortality. Gastric tube placement was more frequent in the thoracotomy group (p = 0.034).
Yalçınkaya et al. (2020) [30]	Thoracotomy vs. video-assisted thoracoscopic surgery (VATs)	A cohort of 13 patients with a single esophageal leiomyoma, except one. Initially, four were scheduled for thoracotomy and nine for VATS. Enucleation was successfully completed in five cases, with the other four having to be converted to thoracotomy (one lack of surgical experience in the first patient of this cohort, two mucosal injuries, and one intraoperative difficulty in tumor localization). Two of these four patients went on to develop complications postoperatively (one postoperative bleeding and one prolonged air leak) and required re-thoracotomy. Mean hospitalization 5.5 days (range: 3-7 days). Only one patient had complications during the follow-up. All 13 had no recurrence.
Rege et al. (2023) [31]	Laparoscopic transhiatal enucleation	No postoperative iatrogenic mucosal injury reported. No conversions or major complications postoperatively, such as postoperative leak or death. Complete recovery with patients tolerating sips at six hours postoperatively and being able to be discharged on a liquid diet once tolerated. Patients graduated from a full diet two weeks postoperatively after a one-week clinic follow-up. At three-month follow-up, two patients reported GERD responsive to PPI. The rest of the 19 cases were asymptomatic. Complete laparoscopic resection. Laparoscopic excision of the tumor expedited postoperative recovery of all our patients compared to the traditional approaches of thoracotomy.
Kemuriyama et al. (2021) [32]	Comparison of video-assisted thoracoscopic surgery (VATS) vs. robotic-assisted thoracoscopic surgery (RATS)	RATS resulted in 0% mucosal injury compared to 1-15% with VATS and open surgery. RATS led to a shorter hospital stay (1–7 days, mode: 3.5 days) compared to VATS (2–10 days, mode: 6 days). No serious complications with RATS, whereas VATS had reported complications including mucosal injury, fistula formation, and atelectasis.
Li et al. (2024) [36]	Piecemeal submucosal tunneling endoscopic resection (P-STER)	16 patients underwent P-STER with only one patient experiencing postoperative complications (esophageal fistula, treated conservatively). The mean length of stay was 11.81 ± 7.30 days. No recurrence or complications during follow-ups.
Chen et al. (2022) [37]	Robotic-assisted thoracoscopic (RATS) enucleation	A total of 19 patients underwent robotic-assisted thoracoscopic enucleation of esophageal tumors. The mean tumor/cyst size was 55 mm (15–220 mm). Two cases were shifted to minimally invasive esophagectomy (10.5%) due to intraoperative pathological confirmation of malignant gastrointestinal stromal tumors with mucosal invasion. Perioperative complications occurred in three (15.8%) cases, without 30-day surgical mortality. There was no recurrence of tumor or symptoms in a mean follow-up of 35 months. Median operation time was 99 minutes (range = 71–247 minutes), with minimal blood loss (increased loss noted in esophagectomy cases). Median hospital stay was 11 days (range = 5–53). Postoperative complications included one case of sepsis, another with chylothorax, and a third with GI bleeding, hiatal hernia, and stroke. The median interval between surgery and liquid intake was five days, with no long-term complications observed during the follow-up period.
Tan et al. (2015) [38]	Comparison of video-assisted thoracoscopic surgery (VATS) vs. submucosal tunneling (STER)	Evaluated 31 patients, with 18 undergoing STER and 13 receiving VATS. STER resulted in shorter operation time (75.00 ± 27.17 minutes vs. 123.46 ± 50.18 minutes, p = 0.002), less hemoglobin level decrease (3.54 ± 1.81 g/L vs. 9.33 ± 3.64 g/L, p < 0.001), shorter hospital stays (6.00 ± 1.19 days vs. 8.85 ± 2.64 days, p < 0.001), and lower cost (USD 3379.4 ± 702.8 vs. USD 4614.7 ± 862.3, p < 0.001). En bloc resection was achieved in 88.9% (16/18) in the STER group and 100% (13/13) in the VATS group (p > 0.05). No recurrence was noted in either group during follow-up (mean: STER 18.94 ± 8.19 months; VATS 38.77 ± 18.15 months, p < 0.001). Complications were comparable between groups (STER: 16.7%, VATS: 15.4%, p > 0.05).
Wang et al. (2020) [39]	Submucosal tunneling endoscopic resection (STER)	Evaluated 24 patients who underwent STER for esophageal leiomyoma. The mean tumor size was 38 mm (range: 15–62 mm). Complete en bloc resection was achieved in all cases. Mean operative time: 65 minutes. No conversions to open surgery. Complications included intraoperative pneumoperitoneum (one case), postoperative pneumothorax (two cases; one case spontaneously resolved, one case required thoracic drainage), minimal bilateral pleural effusion (three cases; spontaneously resolved), and minor mucosal injury (seven cases; intraoperatively repaired with hemostatic clips), all managed conservatively. Postoperatively, some patients had CT selectively. Mandatory 24-hour fasting after procedure, followed by a liquid diet for 24 hours before being graduated to a soft-food diet for two weeks. Mean hospital stay: 4.2 days. No recurrence was observed at three, six, and 12-month follow-ups to endoscopically assess wound healing and evaluate for residual tumor or recurrence. STER was associated with minimal invasiveness, no major complications, low morbidity, and fast recovery.
Pham et al. (2022) [40]	Minimally invasive surgery (MIS) using video-assisted thoracoscopic surgery (VATS) or video-assisted laparoscopic surgery (VALS)	Evaluated feasibility and outcomes in 75 patients. VATS was performed in 52 cases, while VALS was preferred for tumors near the gastroesophageal junction (23 cases). Mean operative time: 105 minutes (VATS) and 174 minutes (VALS). Mucosal injury occurred in 6.7% of cases, all repaired without long-term complications. Mean hospital stay: 7.2 days. No major perioperative complications or mortality. Seven patients developed GERD requiring PPIs. No long-term esophageal stenosis, recurrence, or malignancy observed.
Claus et al. (2013) [41]	Thoracoscopic enucleation in the prone position	10 cases were completed in the prone position without conversion. Mean operating time: 89.2 ± 28.7 minutes. Bleeding was negligible, and no intraoperative or postoperative complications occurred. No ICU support was needed. Postoperative chest X-ray showed no significant changes, with two cases of minor atelectasis. Minimal chest tube drainage and air leak in all 10 cases, with tube removal at 24 hours. Mean hospital stay: 3.2 days. Contrast X-ray at three months showed no abnormalities. No recurrence or complications at one- and three-month follow-ups. Thoracoscopy in the prone position allowed for optimal visualization and reduced postoperative discomfort compared to thoracotomy.
Aydin et al. (2013) [42]	Thoracoscopy vs. thoracotomy for esophageal leiomyoma enucleation	In a cohort of eight patients, three patients underwent thoracoscopic enucleation and five had thoracotomy (two right thoracotomies, two left thoracotomies, and one laparotomy and enucleation). No immediate postoperative complications. Patients were discharged on postoperative day eight (mean: day 8; range: 5-12 days). However, one of the thoracoscopic enucleation patients was noted to have a pseudodiverticulum at the three-month follow-up. No recurrence on follow-up.
Priego et al. (2006) [43]	Thoracotomy vs. thoracoscopy	In a cohort of nine patients, five patients underwent an open approach (three thoracotomies and two laparotomies) and four patients underwent an endoscopic approach (two thoracoscopies and two laparoscopies). Mean operating time was 182.14 minutes (open: 146.25 minutes vs. endoscopic: 230 minutes). All patients had an uneventful postoperative period, with the endoscopic group being discharged on postoperative day 3.25 compared to the open group, which was discharged on postoperative day seven. Long-term, two patients in the endoscopic group required reoperation for reflux symptoms and developed post-surgical thoracic pain and were found to have intestinal adhesions eight years postoperatively.

Case Reports and Rare Presentations

The case reports highlighted unique presentations of esophageal leiomyomas, including a case of sudden asphyxia due to airway compression, giant tumors requiring esophagectomy, and tumors mimicking malignancies on imaging. Rare cases included an asymptomatic 20-year-old female with an incidental ultrasound finding and a 37-year-old male who developed an esophagopleurocutaneous fistula postoperatively. Most patients recovered well post surgery, with no recurrence at follow-up (Table 5).

**Table 5:** Case reports and rare presentations of esophageal leiomyoma. CT: computed tomography; PET-CT: positron emission tomography-computed tomography; EUS: endoscopic ultrasonography; EUS-FNA: endoscopic ultrasonography with fine needle aspiration; RATS: robotic-assisted thoracoscopic surgery; NG: nasogastric; COD: cause of death; SMA: smooth muscle actin; GERD: gastroesophageal reflux disease; NPO: nothing per mouth; EGD: esophagogastroduodenoscopy.

Study	Patient demographics	Symptoms	Imaging findings	Approach	Outcome
Kharlamova & Zhayvoronok (2024) [14]	20-year-old female	Asymptomatic, incidental finding on screening ultrasound	Barium swallow: round filling defect measuring 25 x 35 mm. EGD: smooth, spherical, submucosal mass in the upper esophagus protruding into the esophageal lumen with no changes to the overlying mucosa.	N/A	N/A
Kramer et al. (1986) [12]	37-year-old male	Vague chest discomfort for several years	Chest X-ray: large paracardial mass in the left hemithorax.	Exploratory thoracotomy	The patient developed esophagopleurocutaneous fistula postoperatively, which did not resolve with chest tube drainage. Later, the patient also developed bright red blood in the NG tube. Esophagoscopy revealed active bleeding in the distal esophagus secondary to a large leiomyoma and a persistent esophagopleural fistula. The patient was managed surgically with esophagectomy. The patient was discharged on postoperative day 41. No complications or recurrence at follow-ups over the next 6 years.
Schluger et al. (1956) [9]	47-year-old male	Suprapubic abdominal pain, anorexia, and weight loss > 36 lbs x 4 days	Barium swallow: sharply defined, persistent filling defect on the right anterolateral wall esophageal wall. Chest X-ray: well-circumscribed, submucosal filling defect in the middle third of the esophagus, attached to the anterior esophageal wall.	Exploratory thoracotomy	A 13 x 35 x 2 cm mass was completely resected intraoperatively. No postoperative complications or recurrence. The patient had a fever on postoperative day one and was given antibiotics. Chest tube removed, patient ambulatory and liquid diet tolerated postoperative day one. Graduated to soft diet and then regular diet on postoperative days two and five, respectively, before being discharged on day 16.
Peacock et al. (1985) [10]	26-year-old female	Dyspnea, loss of consciousness	N/A	N/A	Unsuccessful resuscitation. COD: sudden asphyxial death due to an esophageal leiomyoma. Post-mortem findings: 7.5 cm diameter, firm, grayish-white, well-defined submucosal mass attached to the anterior wall of the upper, middle esophagus. The tumor compressed the posterior trachea above the bifurcation, partially occluding the tracheal lumen, which was found to be hemorrhagic at the constriction point.
Elbawab et al. (2021) [16]	24-year-old male	Dyspnea, productive cough, dysphagia	Chest X-ray: mediastinal mass >8 cm in width. CT: multilobulated mass originating from the upper, middle esophagus, causing severe esophageal lumen narrowing and compression of the adjacent trachea and right main bronchus.	Esophageal resection	No complications or recurrence postoperatively. Admitted to ICU postoperatively, extubated on day two, and downgraded to wards. Discharged on day 10 following negative gastrografin contrast study showing no leaks. Asymptomatic at one week, two weeks, and one-year clinic follow-ups.
Rao et al. (2020) [19]	35-year-old female	Hiccups and belching for >30 years, recent choking sensation	Gastroscopy: uplifted lesion in the cardia. CT: space-occupying lesion, suspected malignancy. PET-CT: increased metabolism in the lower esophagus, malignancy suspected. EUS: hypoechoic mass (49 mm), irregular margins, internal vascularity, close to liver and diaphragm.	EUS-FNA was performed preoperatively due to suspicion of a stromal tumor. Leiomyoma confirmed using immunohistochemistry and managed surgically with laparoscopic resection.	Leiomyoma confirmed by postoperative pathology. Complete resection, no complications. Complete recovery.
Karagülle et al. (2008) [29]	52-year-old male	Dyspepsia and esophageal reflux	Chest X-ray: right hemithorax mass. Esophagography: filling defect. CT: giant calcified 110 x 99mm mass in the distal esophagus invading into the surrounding cardia and fundus of the stomach. EUS: 90-150 mm diameter mass between the carina and the cardia	Right thoracotomy and midline laparotomy with distal esophagectomy and esophagogastrostomy.	Complete surgical resection achieved. Discharged on post-op day six. Follow-up at 25 months showed mild erosive esophagitis at the anastomosis line, but no recurrence.
Kemuriyama et al. (2021) [32]	Female in her 30s	Epigastric pain	Endoscopic ultrasound-guided fine-needle aspiration suggested benign leiomyoma, but the size (100 mm) indicated malignant potential.	Robot-assisted thoracoscopic surgery (RATS) with the da Vinci Xi system.	Successful enucleation; operation time: five hours and 29 minutes; blood loss: 21 mL; no complications, no mucosal injury; discharged on post-op day four.
Elliott et al. (2021) [33]	69-year-old male, former smoker, in good overall health	Vague epigastric discomfort, occasional sensation of food sticking in chest	Upper endoscopy: subepithelial lesion in mid-esophagus. EUS: 24 × 17 × 16 mm mass from muscularis propria, confirmed as spindle cell neoplasm (likely leiomyoma). Repeat EUS one year later showed a stable 18 × 17 mm submucosal mass. CT: smooth mid-esophageal mass centered in the left posterior wall.	Robotic-assisted thoracoscopic enucleation with four robotic ports. Circumferential esophageal dissection performed, mass enucleated, muscle closed with 3–0 Vicryl sutures.	Complete recovery with no postoperative complications. Esophagram on postoperative day one showed no perforation. Started on clear liquid diet, discharged on postop day two. Pathology confirmed leiomyoma (2.3 cm, spindle cells, positive for SMA and caldesmon).
Everitt et al. (1992) [44]	48-year-old female	Epigastric discomfort and heartburn x 3 years; dysphagia to solids, retrosternal pain, 4 kg weight loss x 6 months	Barium swallow: sharply defined, persistent filling defect in the middle esophagus. Esophagoscopy: 20-mm mass attached to the right posterior wall of the esophagus 250 mm down its length with normal overlying mucosa.	Thoracoscopic enucleation	Uneventful postoperative period. The patient reported reduced dysphagia postoperatively.
Oyama et al. (2020) [45]	42-year-old female	Postprandial abdominal pain x 6 months	CT: >60 mm, complicated tumor with slightly enhanced in the delayed phase on enhanced CT surrounding the lower esophagus. EGD: submucosal tumor with a sub-circumferential elevated lesion near the esophagogastric junction. EUS: irregularly shaped tumor originating from the muscularis mucosa layer surrounding 3/4 of the lower esophagus circumferentially.	STER and thoracoscopic enucleation for treatment of large, complicated esophageal leiomyomas.	Complete operation time was 273 minutes (endoscopic: 68 minutes; thoracoscopic: 185 minutes) with a total blood loss of 25 g. On postoperative day two, the patient had an esophageal endoscopy confirming the absence of leaks or stenosis before resuming oral feeds on postoperative day three. The patient was discharged on postoperative day seven with no complications. No tumor recurrence or long-term complications on follow-up.
Pacheco-Barzallo et al. (2011) [46]	49-year-old female	Progressive dysphagia to solids, epigastric abdominal pain, halitosis, GERD x 10 months	EGD: 25 mm diameter, intraluminal filling defect of the esophagus, a few centimeters from the cardia. EUS: hyperechoic, sharp-margined tumor with normal overlying mucosa	Laparoscopic enucleation	Uneventful postoperative period with the patient resuming oral feeds once esophagram showed flow of contrast to the stomach, with the patient being discharged on postoperative day five. No complications or recurrence on follow-up.
Gadelkarim et al. (2022) [47]	53-year-old obese male	Progressive dysphagia to solids and odynophagia x 1 year	Barium swallow: lobulated mass causing extrinsic compression and luminal narrowing/filling defect in the middle esophagus. CT: mildly dilated upper esophagus with soft-tissue mass arising distal to the dilation arising from the middle esophageal wall at the level of the esophagus.	Robotic-assisted thoracoscopic enucleation	Uneventful postoperative course. Postoperatively, oral diet was resumed after a negative barium swallow for esophageal leaks. The patient was discharged on postoperative day four with no recurrence or complications at two-week follow-up.
Feng (2023) [48]	55-year-old male	Dysphagia x 3 years	Chest X-ray: right-sided displacement of the esophagus, with narrowing of the lower thoracic esophagus and dilatation of the upper esophagus. CT: 70 x 50 mm large, soft-tissue, heterogeneously enhanced mass with bulky calcifications and clear margins arising from the posterior mediastinum at the level of T9/T10.	Video-assisted thoracoscopic surgery (VATS)	Uneventful postoperative course. No postoperative complications or recurrence.
Gandhi et al. (2022) [49]	37-year-old male	Dysphagia	EUS: 65 x 25 x 40 mm mass arising from the muscularis propria layer of the esophagus.	Submucosal tunneling endoscopic resection (STER)	Operative time: 210 minutes. Uneventful postoperative period with the patient being kept NPO for two days before resuming oral feeds on postoperative day three. The patient was discharged without incident on postoperative day six with no complications or recurrence at four-week follow-up.
Higuchi et al. (2021) [50]	38-year-old man	Dysphagia x several months	Barium swallow: smooth, rounded filling defect in the lower third of the esophagus. EGD: enlarged, protruding, submucosal mass and luminal narrowing in the lower third of the esophagus. CT: 90 x 40 mm circumferential mass.	Thoracoscopic enucleation	Operative time: 288 minutes with minimal blood loss (14 ml). On postoperative day four, the patient resumed oral intake before being discharged on postoperative day seven.
Khalaileh et al. (2013) [51]	40-year-old male	Asymptomatic, incidental finding on screening X-ray	Chest X-ray: lower mediastinal mass. Barium swallow: filling defect in the lower esophagus with intact mucosal surface. CT: 40 x 30 mm mass in the distal esophagus. EGD: submucosal mass protruding into intraluminal space with intact overlying mucosa. EUS: 50 x 30 mm mass with no lymphadenopathy.	Robot-assisted thoracoscopic enucleation	Lesion enucleated without mucosal penetration. Intraoperative endoscopy confirmed mucosal integrity. Operative time: 288 minutes, with minimal blood loss (blood loss: <20 mL). Uneventful postoperative course. Gastrografin swallow study on post-op day one showed good esophageal clearance and no leak. Discharged on post-op day three, tolerating a liquid diet. Pathology confirmed a benign leiomyoma.
Elkholy et al. (2021) [52]	42-year-old male	Dysphagia	N/A	Submucosal tunneling endoscopic resection (STER)	Complete separation of the 3.3 x 1.7 cm tumor with no complications or recurrence.
Ivey et al. (2020) [53]	53-year-old female	Epigastric pain, dysphagia, constipation, and a history of esophageal hiatal hernia	CT: 37 x 30 mm esophageal leiomyoma	Robot-assisted thoracoscopic enucleation (RATS)	Successful resection of the tumor with minimal blood. The procedure was completed in 155 minutes. The patient was discharged on postoperative day two on a liquid diet. No complications or recurrence at six-week follow-up.

Discussion

Current Guidelines on the Management of Esophageal Leiomyoma

Esophageal leiomyoma management depends on tumor location, size, symptom severity, comorbidities, and patient condition. According to the ESGE guidelines, EUS is the most accurate tool for characterizing subepithelial esophageal lesions, including leiomyomas, as it enables assessment of size, location, originating layer, echogenicity, and shape. However, since EUS alone cannot distinguish all differentials, adjunct imaging such as X-ray, barium swallow, CT, and MRI may be required for comprehensive evaluation.

For small, asymptomatic leiomyomas, ESGE does not recommend routine surveillance if the diagnosis is definitive. However, for asymptomatic subepithelial lesions without a clear diagnosis, ESGE suggests esophagogastroduodenoscopy (EGD) surveillance at three to six months initially, followed by two to three-year intervals for lesions <10 mm and one to two-year intervals for lesions measuring 10-20 mm. For subepithelial lesions >20 mm that are not resected, ESGE suggests EGD plus EUS at six months, then at six to 12-month intervals. Of the subepithelial lesions originating in the muscularis propria layer of the esophagus, 98% have been confirmed to be leiomyomas based on histology.

Surgical intervention is recommended for symptomatic patients, tumors exceeding 5 cm, increasing size on surveillance, and suspected malignancies [18]. The treatment strategy should be determined based on tumor size and accessibility and include surgical options like enucleation and esophageal resection, with enucleation being the preferred method due to its efficacy and lower recurrence rate. Endoscopic full-thickness resection (EFR) is considered an option but is typically limited to tumors ≤35 mm to allow en bloc removal. When resection is necessary, ESGE recommends STER over an exposed full-thickness resection to minimize complications. In cases where endoscopic removal is not feasible, thoracoscopic enucleation remains the preferred surgical approach due to its efficacy and low recurrence rate [15,17,30,41].

While the ESGE guidelines endorse endoscopic evaluation and surveillance, ESGE recommends EUS for assessing subepithelial lesions and endoscopic resection for symptomatic cases [2,20]. The Society of Thoracic Surgeons (STS) suggests a conservative approach for asymptomatic leiomyomas and favors minimally invasive techniques like VATS when surgery is necessary [21-23,30]. The American Society of Clinical Oncology (ASCO) emphasizes the importance of differentiating leiomyomas from GISTs and advocates surgical resection for symptomatic or large leiomyomas, with endoscopic options considered for appropriate cases [30,41]. The American Society for Gastrointestinal Endoscopy (ASGE) supports the use of endoscopy and EUS for diagnosis and management, recommending endoscopic treatment for symptomatic lesions and regular surveillance for asymptomatic ones [2,25,34].

For larger or more complex leiomyomas, particularly those with extensive mucosal adhesion or located at the gastroesophageal junction, more aggressive management strategies are required. Segmental or total esophagectomy via either a thoracic or thoracoabdominal approach is the preferred surgical method for giant leiomyomas, diffuse leiomyomatosis, circular tumors, and tumors densely adhesive to the mucosa [12,17,29]. For lesions presenting in the region of the gastroesophageal junction, laparotomy is used [23]. Tumors in the lower third of the esophagus may also require an anti-reflux procedure in conjunction with enucleation or resection [24]. EUS and FNA play crucial roles in diagnosis and staging [18,19,40].

Advantages of Thoracoscopic Enucleation

Open enucleation via right thoracotomy is standard for tumors in the upper and middle thirds of the esophagus [22,23,41]. Enucleation via oral endoscopy is possible for small, pedunculated tumors [41]. Laparoscopic enucleation is preferred for tumors in the lower third and for patients with lung dysfunction [11,22].

Minimally invasive thoracoscopic surgical enucleation methods, including VATS and RATS, offer shorter hospital stays, reduced postoperative pain and infection rates, and minimal aesthetic compromise compared to open thoracotomy [2,6,39]. VATS, as detailed by Luh et al. (2012), allows for precise tumor removal while reducing hospital stays and postoperative pain, particularly in patients with tumors located in the upper and middle thirds of the esophagus, where direct visualization and manipulation are critical [11]. RATS, on the other hand, provides superior benefits through its three-dimensional view and enhanced magnification, which helps minimize mucosal perforation and allows for more delicate dissection [51]. This technique has demonstrated improved outcomes in terms of reduced complications and better visual control of the surgical field.

Thoracoscopic enucleation, compared to traditional thoracotomy, offers significant benefits in terms of postoperative recovery and complication risk reduction. Multiple studies have demonstrated that VATS is associated with shorter hospital stays and reduced postoperative pain. Choi et al. (2011) reported that patients undergoing VATS had a significantly shorter hospital stay (8.0 vs. 10.7 days, p = 0.006) compared to those who underwent thoracotomy, with minimal complications and no perioperative mortality. Similarly, A-Lai et al. (2022) found that thoracoscopic enucleation resulted in smaller incisions, reduced trauma, and decreased postoperative pain, with a lower frequency of gastric tube placement compared to thoracotomy (P = 0.034) [28]. Yalçınkaya et al. (2020) further highlighted that while some patients initially scheduled for VATS had to be converted to thoracotomy due to intraoperative difficulties, the overall complication rate remained low, and hospitalization duration was relatively short (mean: 5.5 days) [30].

Similarly, RATS, while still relatively new, has demonstrated superior precision and reduced complications, particularly in preventing mucosal injury and postoperative complications such as fistula formation and atelectasis [32]. RATS has been associated with minimal morbidity, with studies reporting 0% mucosal injury compared to 1-15% in VATS and open surgery, as well as shorter hospital stays (mode: 3.5 days for RATS vs. six days for VATS) [27,32,37,53]. While each technique has its own indications, careful patient selection remains critical to optimizing surgical outcomes. Therefore, traditional surgical enucleation, while effective, carries a higher risk of morbidity, prolonged hospital stays, and increased complications compared to these newer techniques [28,39].

Advantages of Minimally Invasive Resection Techniques

The evolution of minimally invasive techniques has led to improved outcomes in esophageal leiomyoma management, offering reduced complications, shorter hospital stays, and lower morbidity compared to traditional surgical enucleation [3,14,28,31,36,40]. Minimally invasive resection methods for esophageal leiomyomas include STER, endoscopic submucosal tunneling dissection (ESTD), endoscopic full-thickness resection (EFR), and endoscopic submucosal excavation (ESE). These techniques are valuable for treating various subepithelial lesions, providing flexibility in surgical approach depending on the tumor's characteristics, such as tumor size, depth, and origin [39].

Minimally invasive techniques such as STER have been shown to further optimize surgical outcomes, demonstrating excellent safety and efficacy. Tan et al. (2015) reported that STER resulted in shorter operative times (75.00 ± 27.17 minutes vs. 123.46 ± 50.18 minutes, p = 0.002), less hemoglobin level decrease, and shorter hospital stays (6.00 ± 1.19 days vs. 8.85 ± 2.64 days, p < 0.001) compared to VATS [38].

Studies by Yalçınkaya et al. (2020) demonstrated the favorable outcomes of these techniques, showing that they offer significant benefits, including reduced postoperative pain, shorter hospital stays, and faster recovery times compared to traditional surgical methods [30]. These minimally invasive approaches enhance treatment efficacy while minimizing patient discomfort and recovery time.

Comparison of Minimally Invasive Resection Techniques

ESTD, a technique similar to STER, also involves submucosal dissection and is particularly useful for tumors confined within this layer. ESTD enables precise tumor removal with minimal invasiveness [41]. In contrast, EFR is employed for tumors that extend through the full thickness of the esophageal wall, ensuring complete resection and reducing recurrence risk. While ESE focuses on removing the tumor along with a surrounding margin of submucosal tissue, making it suitable for localized tumors that do not extensively involve deeper layers or adjacent structures.

For leiomyomas originating from the muscularis mucosa, endoscopic mucosal resection (EMR) and ESTD are preferred due to their lower complication rates and shorter recovery periods. According to Sun et al. (2017), patients undergoing EMR/ESTD experienced significantly fewer adverse effects than those treated with STER/ESE, including lower intraoperative bleeding rates, fewer intraoperative perforations, shorter hospital stays (3 ± 2 days in EMR/ESTD vs. 6 ± 2 days in STER/ESE), and faster resumption of diet (1 ± 1 day in EMR/ESTD vs. 4 ± 3 days in STER/ESE) [6].

For leiomyomas from the muscularis propria, more advanced endoscopic and surgical techniques are necessary. STER, which involves creating a submucosal tunnel to access and remove the tumor, is highly effective for larger submucosal tumors (>10 mm) while minimizing disruption to surrounding tissue, leading to quicker recovery [38]. Elkholy et al. (2021) described a case where STER was used successfully by creating a submucosal bleb 5 cm proximal to the lesion, tunneling through the muscle, and removing the tumor before sealing the opening with endoclips [52]. This method offers precise tumor removal with minimal trauma to the esophageal wall.

Additionally, novel approaches such as thoracoscopic enucleation using a balloon-mounted intraluminal endoscope have shown promising results in facilitating precise tumor removal while minimizing trauma to surrounding tissues [37]. The use of the prone position in thoracoscopic enucleation further enhances surgical outcomes by offering better exposure and access to the tumor site compared to the traditional lateral decubitus position [17]. Proper closure of the myotomy, particularly reapproximating the longitudinal muscles after enucleation, is essential in preventing pseudodiverticulum formation, further improving surgical outcomes [41].

Limitations

Studies that examined leiomyoma in the context of co-existing carcinoma were excluded from this review to mitigate potential bias, as their management approaches often prioritized resection, the standard of care for carcinoma. However, this may not be the optimal approach for isolated leiomyomas. The overlap in management strategies between these two distinct pathologies posed a challenge to our comprehensive discussion of management options for esophageal leiomyoma. Moreover, the current standard of care for esophageal leiomyoma and carcinoma, which emphasizes resection as the primary treatment option, may have limited our exploration of alternative management options. Future research should strive to delineate the management of leiomyoma and carcinoma to provide a more nuanced understanding of treatment options for these discrete pathological entities.

## Conclusions

In conclusion, this systematic review provides a comprehensive overview of the current treatment options for esophageal leiomyomas, highlighting the evolving landscape of management strategies for this rare and benign tumor. The evidence suggests that minimally invasive interventions, such as STER and RATS, offer improved outcomes and reduced complications compared to traditional surgical enucleation. As these approaches continue to evolve, they may become the new standard of care, providing improved outcomes, reduced complications, and shorter hospital stays. However, further studies are needed to fully establish their efficacy and long-term outcomes. Moreover, future research should aim to clearly distinguish between the management of leiomyoma and carcinoma, providing a more nuanced understanding of treatment options for these distinct pathological entities. By synthesizing the available literature, this review informs evidence-based practice and guides future research in the management of esophageal leiomyomas, ultimately improving patient care and outcomes.
